# Energetic characterization and radiographic analysis of torrefied coated MDF residues

**DOI:** 10.1038/s41598-021-84296-5

**Published:** 2021-03-01

**Authors:** Paula Gabriella Surdi de Castro, Vinícius Resende de Castro, Antonio José Vinha Zanuncio, José Cola Zanuncio, Angélica de Cássia Oliveira Carneiro, Jorge Gominho, Solange de Oliveira Araújo

**Affiliations:** 1 Departamento de Engenharia Florestal, Universidade Federal de Viçosa, Viçosa, 36570-900 Brazil; 2 Instituto de Ciências Agrárias, Universidade Federal de Uberlândia, Monte Carmelo, 38500-000 Brazil; 3 Departamento de Entomologia/BIOAGRO, Universidade Federal de Viçosa, Viçosa, 36570-900 Brazil; 4 Centro de Estudos Florestais, Instituto Superior de Agronomia, Universidade de Lisboa, 1349-017 Lisboa, Portugal

**Keywords:** Renewable energy, Bioenergy

## Abstract

The use of wood panel residues as biomass for energy production is feasible. Heat treatments can improve energy properties while minimizing the emission of toxic gases due to thermoset polymers used in Medium Density Fiberboard (MDF) panels. Torrefaction or pre-carbonization, a heat treatment between 200 and 300 °C with low oxygen availability accumulates carbon and lignin, decreases hygroscopicity, and increases energy efficiency. The objective of this work was to evaluate the energy parameters (immediate, structural, and elementary chemical composition, moisture content, and yield) and density in torrefied MDF panels. The torrefaction improved the energetic features of coated MDF, decreasing the moisture content, volatile matter, and consequently, concentrating the carbon with better results in the samples torrefied for 40 min. The densitometric profiles of the torrefied MDF, obtained by X-ray densitometry, showed a decrease in the apparent density as torrefaction time increased. The digital X-ray images in gray and rainbow scale enabled the most detailed study of the density variation of MDF residues.

## Introduction

World production of reconstituted wood panels, such as Medium Density Fiberboard (MDF), and of High Density Fiberboard (HDF), was 99.5 million m^3^ in 2018^1^, with Brazil being the eighth largest world producer with 8.2 million m^3^ and a 2.5% increase in panel production of medium density fibers (MDF) compared to the previous year^2^. The demand increase for MDF panels raises problems with the generation of residues associated with the production process^3^, which can be used as a source of biomass for energy production^4–6^.

Woodworking (main use of melamine-coated MDF) and furniture factories in series (main use of uncoated raw MDF) accumulate residues from wood panels until they are sent to steam and energy generation boilers or deposited in inappropriate areas^7^. These operations are difficult to perform due to synthetic resins based on urea–formaldehyde or phenol–formaldehyde, which are thermoset polymers of the panels^7–9^.

A layer of melamine (low pressure) covers one or both faces of MDF panels, used in woodworking, to simulate the appearance of a natural wood product^10^. Edge tapes often hide the core of the melamine-coated panels in a process whose efficiency depends of the surface finish^11^. This process generates residues that must be burned in a controlled environment, as they release formaldehyde and VOC's (volatile organic components) into the atmosphere, such as toluene and limonene, which can cause respiratory syndromes and are considered carcinogenic^7,12^.

Torrefaction, carried out with controlled temperatures and low oxygen availability, aims to concentrate carbon and lignin in the material^13–15^. The MDF panel torrefaction can eliminate gaseous products derived from nitrogen compounds, such as HCN and NOx during combustion; and NH_3_, HNCO, and HCN during pyrolysis. Varnishes and adhesives, such as urea, melanin, and formaldehyde, common in wood panel residues, can be the origin of these compounds^16,17^.

The quality of the torrefied material depends on its energy potential, the presence of cracks, or disintegration during handling, transport and storage^18^.

Micro tomography and X-ray densitometry allows a more detailed internal analysis of different agroforestry products, such as wood^19^, pellets and briquettes^20^, and OSB wood panels^21,22^, and MDF^23^, torrefied or not.

X-ray densitometry, used in odontology, orthopedy, zoology and zootechnology, is a non-destructive method^24^. This method can characterize and evaluate the deterioration of eucalyptus wood by white rot fungi, detect the heartwood-sapwood limits, the effect of forest management on wood properties, annual biomass production and the relation with its anatomical structure^19,22,25^.

The objective of this study was to evaluate the energy properties and the apparent density by X-ray densitometry of MDF panels (Medium Density Fiberboard) coated with melamine on both sides, untreated and torrefied.

## Results and discussion

The holocellulose content of the coated MDF decreased with increasing torrefaction time, varying from 44.89% (untreated) to 37.50% (torrefaction for 40 min) (Table 1).

**Table 1:** Holocellulose (HOLO), total lignin (TL), extractives (EXT), ashes (AC), moisture (MC), gravimetric yield (GY), apparent density (AD), higher heating value (HHV), volatile materials (VM), fixed carbon (FC), carbon (C), hydrogen (H), nitrogen (N), sulfur (S) and oxygen (O) from the untreated and torrefied MDF residues.

Parameter	Untreated	Torrefaction time (min)		
		20 min	30 min	40 min
HOLO (%)	44.89 ± 1.25b	44.93 ± 1.40b	40.82 ± 1.14ab	37.50 ± 3.80a
TL (%)	39.69 ± 0.79a	41.96 ± 1.11ab	47.47 ± 1.00c	52.02 ± 3.62c
EXT (%)	15.42 ± 0.46d	13.11 ± 0.29c	11.70 ± 0.15b	10.48 ± 0.18a
AC (%)	0.91 ± 0.17a	1.06 ± 0.03b	1.08 ± 0.04ab	1.51 ± 0.03c
MC (%)	10.22 ± 0.59c	8.79 ± 0.13b	8.48 ± 0.22ab	8.07 ± 0.47a
GY (%)	100 c	99.45 ± 0.43c	95.67 ± 1.44b	87.59 ± 3.07a
AP(g cm^−3^)	0.63 ± 0.01c	0.61 ± 0.01bc	0.57 ± 0.02ab	0.55 ± 0.04a
HHV(kcal kg^−1^)	4636.0 ± 14.14a	4705.0 ± 29.70a	4733.0 ± 1.41a	4861.5 ± 3.54b
VM (%)	82.69 ± 1.03a	82.98 ± 0.75a	81.05 ± 0.58a	78.27 ± 0.16b
FC (%)	16.40 ± 1.18a	15.94 ± 0.78a	17.90 ± 0.57a	20.22 ± 0.19b
C (%)	49.00 ± 0.14a	48.50 ± 0.00a	49.90 ± 0.00b	50.70 ± 0.00c
H (%)	5.71 ± 0.01ab	5.68 ± 0.04ab	5.75 ± 0.00b	5.62 ± 0.04a
N (%)	3.14 ± 0.06a	3.39 ± 0.01a	3.42 ± 0.31a	3.26 ± 0.07a
S (%)	0.03 ± 0.00b	0.02 ± 0.00b	0.02 ± 0.00ab	0.02 ± 0.00a
O (%)	41.20 ± 0.21c	41.32 ± 0.04c	39.85 ± 0.31b	38.89 ± 0.11a

Means followed by the same letter, per line, do not differ (Tukey p > 0.05).

The reduction in the holocellulose content with the longer torrefaction period is possibly due to the exposure of wood compounds to high temperatures. This, initially, breaks the hemicellulose chains, components with lower thermal stability, and, then, depolymerizing the amorphous zone of the cellulose chains and reducing the contents of these constituents in the material^26–28^.

The total lignin content increased with the torrefaction time, being higher in the intervals of 30 and 40 min, 47.47 and 52.02%, respectively.

The increase in the lignin content with the increase in torrefaction time is due to the high thermal stability of this compound, requiring higher temperatures for its degradation^27^ and justifying its proportional increase with the torrefaction time. The breakdown of the covalent chemical lignin bonds, a compound with a high carbon content^29^, releases large amounts of energy increasing its calorific value, being, therefore, a desirable parameter for energy purposes^18^.

The extractives content decreased with the increase in the torrefaction time, from 15.42 (untreated) to 10.48% in the 40-min treatment.

The decrease in the extractives content with longer torrefaction times is associated to the degradation of polar extractives and polyoses, thermally unstable compounds, which volatize between 130 to 250 °C^30^. The adhesive present in the MDF is also volatilized when exposed to high temperatures^31^. The removal of these materials is, environmentally important, because toxic gases composed of nitrogen, such as HCN, NOx, NH_3_, HNCO, HCN^17^, which would be released into the atmosphere at the time of burning, are also removed. The varnishes and adhesives, such as urea, melanin and formaldehyde, common in wood panel residues can be the origin of these compounds^16^.

The ash content increased with the torrefaction time, being 65.93% higher in the torrefied material during 40 min than with the untreated residues.

The increase in ash content with the torrefaction time is characteristic of biomasses submitted to thermal processes at mild temperatures, but the mineral components remain in the biomass even when subjected to high temperatures, while other components, as hemicelluloses and extractives are degraded^32^. This increase is not desirable, since a higher ash content decreases densification, reduces the energy properties of the biomasses^33^ and increases the frequency of cleaning the boiler^34^.

The hygroscopic equilibrium moisture content varied from 10.22 to 8.07%, with a reduction of these values as the torrefaction time increased. This parameter was 13.99, 17.03 and 21.04% lower in the treatments submitted to torrefaction for 20, 30 and 40 min, respectively, than in the control (untreated).

The reduction in the equilibrium moisture content with lower values in the longer torrefaction times is due to the degradation of cellulose and hemicelluloses, with losses of hydroxyl groups responsible for moisture adsorption^27,35^. This reduction, in the treatments with longer torrefaction times, is desirable for energy purposes, as it reduces the amount of energy used to evaporate the water in the biomass^36^.

The gravimetric yield of the coated MDF decreased by 12.41% in the samples torrefied for 40 min compared to those untreated.

The reduction in the gravimetric yield with the torrefaction time increase with lower value for the samples torrefied for 40 min is due to the chemical degradation of the fiber constituents subjected to the temperature of 300 ºC, mainly of the holocelluloses, with greater mass losses as the torrefaction time increased^6,37^. This reduction in 12.41% can be considered low and varies with the material, as it was 28.28% in the torrefaction of wood chips from *Eucalyptus* spp. at 260 ºC for 20 min in an endless screw roaster^18^.

The apparent density was 12.7% lower in the torrefied material for 40 min than in the untreated samples.

The lower apparent density with the torrefaction time increase is similar to that of the gravimetric yield, and it is due to the MDF mass losses and to the thermochemical degradation of its constituents, volatilized in the treatments with longer torrefaction times, reducing the ratio of mass by volume of the samples and, consequently, the apparent density^38^.

The higher heating value of the residues torrefied for 40 min was 4.86% higher than that of untreated.

The higher heating value of biomasses torrefied for 40 min is due to the volatilization of hemicelluloses and other lower energetic compounds and the proportional increase in the lignin content^33,39^. In addition, the higher heating value is associated with the presence of aromatic lignin rings, and the double carbon bonds in these rings release 518 kJ mol^−1^, higher than the single bonds between carbons, which release 348 kJ mol^−1^^40^. Breaking the double bonds releases 48.8% more energy, increasing the higher heating value of the biomass. The higher heating value is one of the main factors to select the biomass for energy purposes and high values result in a greater amount of energy released, facilitating the operations in the boiler and increasing the power generation capacity^32,39^.

The volatile material content of the torrefied residues for 40 min was 5.34% lower than that of the untreated residues.

The low volatile materials is important because they are rich in oxygen (mainly CO and CO_2_), increasing fixed carbon, the most energetic component in the biomass and, consequently, the higher heating value of the raw material^32,39,41^. The higher volatile materials content increases the speed of burning biomasses due to volatile compounds oxidizing and releasing energy faster than the oxidation of the biofuel fixed carbon^42^.

The fixed carbon of the coated MDF was 23.29% higher in the torrefaction treatment for 40 min than with the untreated sample.

The increase in fixed carbon over torrefaction time is due to the oxygen and hydrogen losses during the degradation of unstable wood compounds and carbon concentration^43^. This is also due to the higher lignin content, a more stable compound with a complex structure and with a lower degradation during carbonization^44^, whose contents are positively correlated with those of fixed carbon. The fixed carbon content can be a parameter to select the biomass destined for combustion^45^. The fuel burning with high levels of fixed carbon and low volatile materials tends to be slower, releasing energy for a longer time for total burning^46^, becoming one of the main advantages in energy supply for the blast furnace in the steel industry^47^.

The carbon content increased with the torrefaction time, being 3.47% higher in the 40-min treatment than in the untreated samples*.*

The increase in the carbon content with the torrefaction time is due to the accumulation of lignin in the material, which has a structure composed of phenyl propane groups joined by C–C and C–O–C bonds and a dense and compact molecular structure^45^. The highest levels of carbon and hydrogen are important for choosing the best energy characteristics of biomass for combustion, pyrolysis or gasification, as they increase energy generation^32^.

The hydrogen content, 5.62 to 5.75%, was similar with the treatment times.

The similar content of hydrogen, between treatments, is important because this chemical element is the one that most contributes to the energy generation due to its higher heating value and greater relation to carbon^32,45^. The elementary composition of biomass is important to evaluate the energy generated by the thermal degradation associated with the enthalpy of carbon and hydrogen^48^.

The nitrogen content was similar between treatments, from 3.14 to 3.42%.

The nitrogen content did not vary between treatments, its content is important because the burning of this chemical element leads to harmful emissions, values above 0.6% are undesirable during combustion because it contributes to the formation of toxic nitrogenous compounds such as NOx, N_2_O, HCN, ammonia, isocyanic and hydrocyanic acid that are harmful to the environment^49^. The average nitrogen content of untreated wood is 0.1%, while that of MDF and MDF panels untreated is 5.4%, decreasing to 2.1 to 2.9% when subjected to temperatures between 250 and 300 ºC^16^. The nitrogen values, higher than those recommended, in all treatments are due to the adhesive used in the production of the panels and the presence of melamine coating.

The sulfur content was lower in the torrefied material for 20, 30 and 40 min than in the untreated one.

The reduction in the sulfur content with the torrefaction time may be associated with the removal of residual sulfur with heat treatment^50^. This reduction is positive because the sulfur released into the atmosphere during combustion is harmful^51^.

The oxygen content decreased with the torrefaction time, being 6.82% lower in 40 min than in the untreated samples.

The decrease in the oxygen content, with a behavior contrary to that of carbon and hydrogen, reduces energy generation because it reduces the calorific value of biomass^52^.

**Figure 1:**
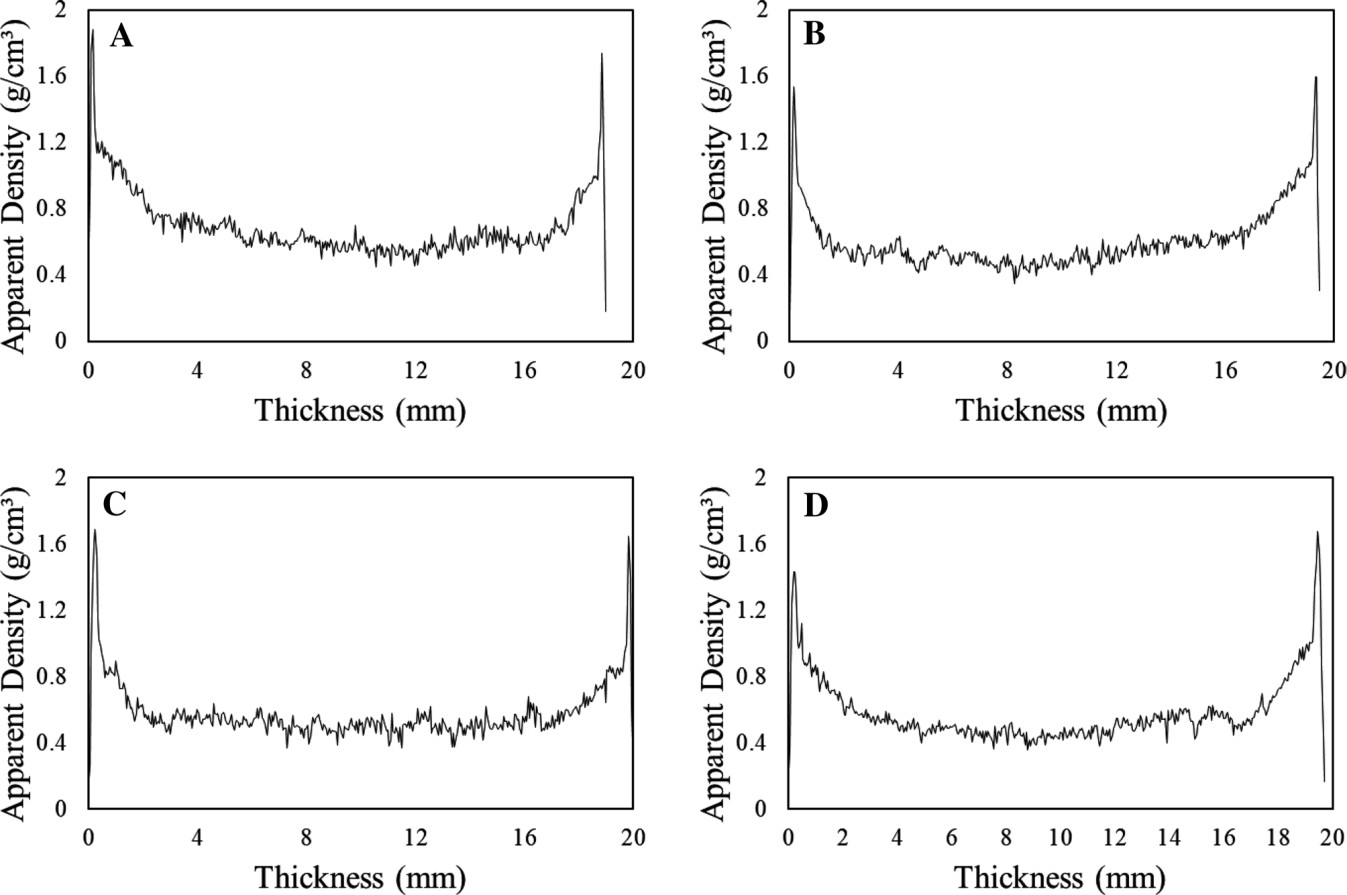
Apparent density profiles (AD) along the thickness of the coated MDF residues. Untreated (**A**); torrefaction for 20 min (**B**); torrefaction for 30 min (**C**); torrefaction for 40 min (**D**).

The panel density was higher at the edges, with lower values in the central region, as assessed by X-ray densitometry (Fig. 1). However, there was a difference between the values of average, minimum and maximum density of MDF panels between the treatments evaluated (Table 2). Finally, the average apparent density in the untreated samples was higher in the evaluation by X-ray densitometry and gravimetric method.

**Table 2:** Apparent density of untreated and torrefied MDF residues for different times.

Torrefaction	Apparent density (g/cm^3^)			
	X-Ray			Gravimetric method
	Average	Maximum	Minimum	
Untreated	0.64 ± 0.02Bc	1.85 ± 0.05cC	0.37 ± 0.12aD	0.63 ± 0.01bD
20 min	0.60 ± 0.01Bb	1.67 ± 0.10cA	0.30 ± 0.11aC	0.61 ± 0.01bC
30 min	0.57 ± 0.01bA	1.67 ± 0.10cA	0.23 ± 0.12aA	0.57 ± 0.02bB
40 min	0.57 ± 0.01bA	1.66 ± 0.04cA	0.27 ± 0.14aB	0.55 ± 0.04bA

Means followed by the same capital letter, per column, or lower case, per line, do not differ by the Tukey test (p > 0.05).

The reduction in the apparent density with the increase in torrefaction time, evaluated with the radiographic method, is similar to that observed with the gravimetric one and it is associated with the reduction of MDF mass due to the thermochemical degradation of its volatilized constituents in treatments with longer torrefaction times^38^.

The density on the MDF panel faces, in all treatments, was high and decreased towards the central region, forming a characteristic profile with a "M" letter shape (Fig. 1). The maximum density, in the upper and lower faces, in all treatments was 1.66 to 1.85 g/cm^3^ (Table 2), due to the thin layer of melamine applied under low pressure on the MDF faces.

The density profiles along the thickness of the MDF panels, with high values on the faces and lower inside, are characteristic of panels made with eucalyptus and pine wood^23,53^. The high values of minimum and average apparent density of the MDF panels are good indicators of high resistance to perpendicular traction and screw pullout, with changes in the density gradient along its thickness, which may affect mechanical strength^54^.

**Figure 2:**
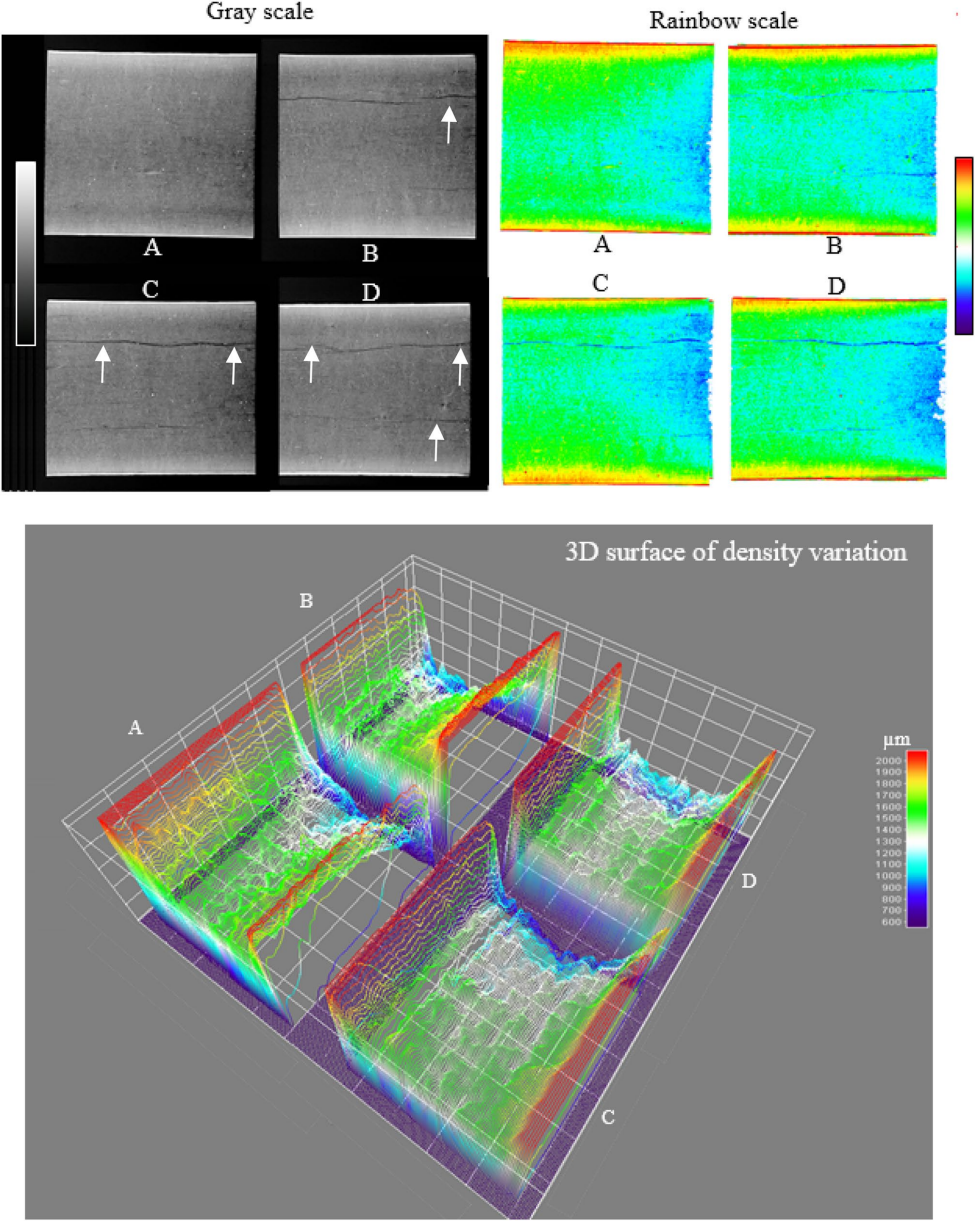
Digital X-ray images of MDF coated residues in gray and rainbow scales and 3D density variation plot. Untreated (**A**); torrefaction for 20 (**B**), 30 (**C**) and 40 (**D**) minutes. Areas with red and blue color indicate higher and lower density, respectively. White arrows indicate the cracks/fissures.

Darkly regions (more central region—core) and more whitish, near to the faces, indicate lower and higher density, respectively, attenuation of the X-ray beams in the reading process, as identified in the analysis of digital X-ray images in the gray scale (Fig. 2). The rainbow scale (colored) with a palette of greater color variability ranging from blue (lower density) to red (higher density) makes easier interpretation and localization of regions with differences in density throughout the sample due to the differentiation of density by color allowing greater contrast compared to gray scale. The torrefaction degraded the intern part of the sample (darker region in the gray scale with a toned color from blue to cyan in the rainbow scale—with lower density), due to the barrier represented by the melamine coating (lighter region in the scale from gray region with toned from yellow to red in the rainbow scale with high density) on both sides of the panel.

The use of X-ray images in 2D and 3D scales with colors gradient makes possible the visualization of non-torrefied regions with a lighter/green color, and, consequently, greater density in the treatment with 20 min of torrefaction. The density increase is due to the insufficient time for the chemical degradation in the center of the panel (core). Increase of the torrefaction period also increased the number of cracks/fissures (Fig. 2). The longer torrefaction time increased the number of cracks/fissures due to greater moisture losses as observed in the MDF and OSB samples pressed at different temperatures^55^.

The 3D surface plot illustrates narrow lines, parallel to each other and perpendicular to the length of the samples, being directly related to the panel density variations (Fig. 2). A low difference between peaks and valleys within the same line implies greater homogeneity in the wood density in that region of the sample. Consequently, the greater their variation throughout the sample, results in greater the fluctuation in the wood density. In addition to the variation of the lines, it was possible to observe the color variations with the blue lines indicating lower density and the red ones greater density. The 3D surface plot provides a better visualization and interpretation of the apparent density variation throughout the sample based on digital X-ray images. The number of peaks and valleys in the same line are directly related to variations in the density of the MDF in the sample region. The distribution of peaks and valleys throughout the MDF, in the samples of control MDF untreated, was homogeneity in the 3D images (Fig. 2A). The samples submitted to thermal treatments had higher variation in this distribution (Fig. 2B,C, D), especially those with longer times, due to thermal degradation of chemical components subjected to high temperature (300 ºC) and residence times. Plotting the 3D density surface by colors and lines of the rainbow scale allowed for greater color contrast and study of the density variation in the 3D scale, compared to the gray scale. The three-dimensional structure of the internal region of the materials studied (MDF) and the increased speed of data analysis confirm satisfactory results, with the use of the 3D scale to study the density of wood and its growth rings and the seed integrity^22,25^. The use of X-ray images, in gray scale or with color gradient in 2D and 3D, are non-destructive and important methods as an additional methodology to the traditional laboratory technological characterization^19,22,25^. The proposed method provides a quick, easy to interpret and reliable solution to assess the apparent density, by X-rays, of torrefied coated MDF residues from digital images.

In conclusion, torrefaction improved the energetic properties of MDF residues, decreasing the equilibrium moisture and volatile materials, and, consequently, the carbon concentration with better results in the torrefied samples for 40 min. The results in the torrefied samples for 30 min showed better values of energetic characterization than 20 min and MDF untreated. The densitometric profiles of the torrefied MDF, obtained by X-ray densitometry, showed a decrease in the apparent density as the torrefaction time increased. The apparent density variation in the digital images of X-ray in gray scale, and rainbow, made possible a more detailed and precise study of the density variation of the MDF residues.

## Methods

### MDF torrefaction

MDF samples with coating (melamine) on both sides, from woodwork disposal, were cut to 18 × 18 × 18 mm pieces, dried in an oven at 103 ± 2 °C to 0% humidity and torrefied for 20, 30 and 40 min at 300 °C.

**Figure 3:**
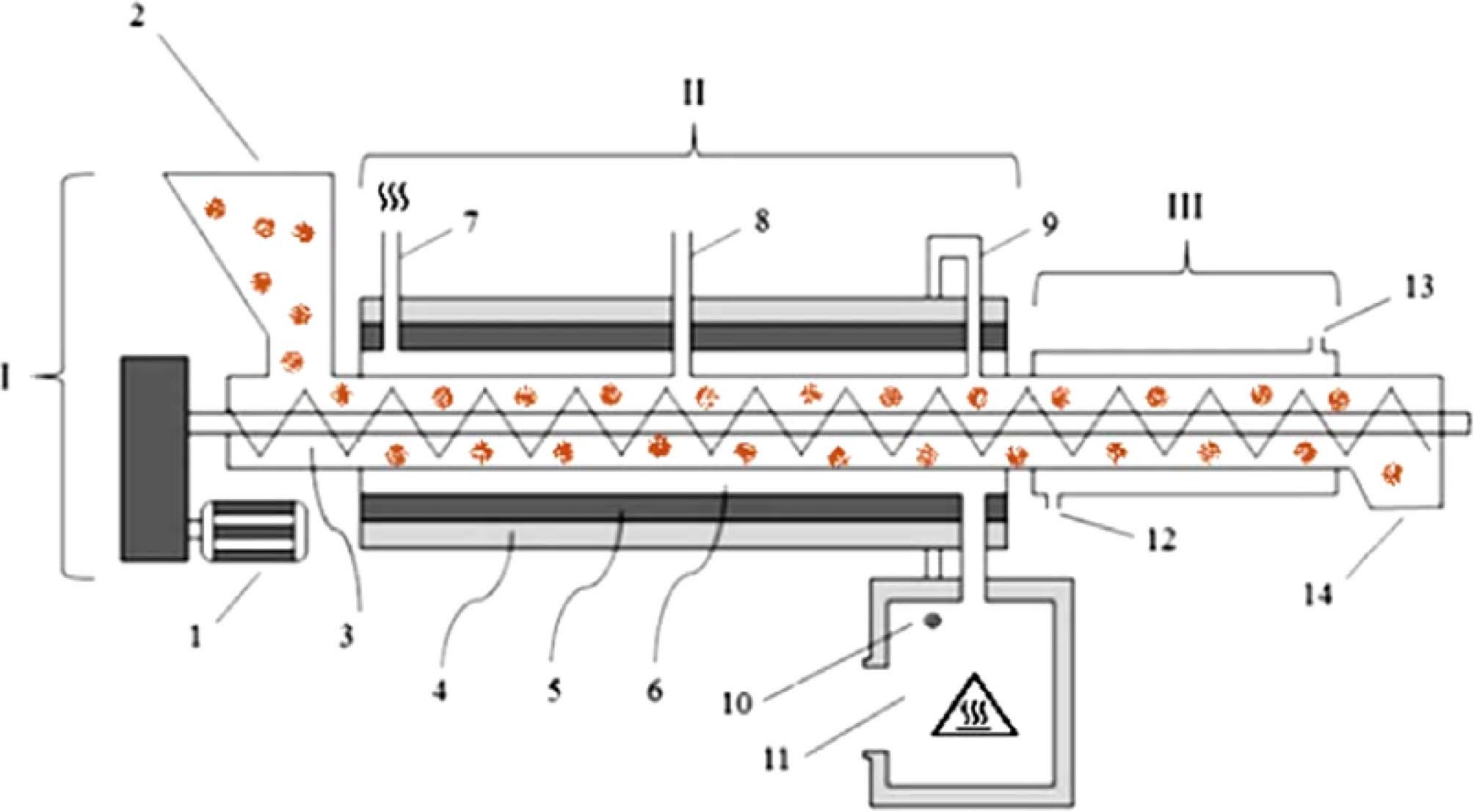
Lateral layout of a prototype screw reactor developed by a Brazilian university for thermal treatment of lignocellulosic biomass, where: I—transport system; II—heating system; III—cooling system; 1—motor; 2—input biomass; 3—worm-screw; 4—insulating layer; 5—refractory layer; 6—flow of heating gas; 7—heating gas output; 8—first "chimney"; 9—second "chimney"; 10—connection "chimney" with the burner; 11—connecting burner; 12—water supply; 13—water outlet; 14—exit of torrefied biomass^18^.

Torrefaction was performed in an endless screw reactor, developed in the Panels and Wood Energy Laboratory of the Federal University of Viçosa, Brazil^14^. The prototype of this equipment was a semi continuous screw reactor, which reuses the volatile gases in the heating system (Fig. 3). The primary structure of this reactor has three essential systems, like most reactors that facilitate dry torrefaction: (I) transport; (II) heating; and (III) cooling. The first system moves the biomass to the heating process, classified as continuous, intermittent or mixed; the second produces and transfers heat to the biomass under controlled conditions for direct or indirect heating; and the third releases the torrefied biomass within a safe temperature limit.

The gravimetric yield of the torrefaction process was calculated by the ratio between the mass of the torrefied material produced and the dry mass of the MDF sample used as input.

### MDF untreated and torrefied properties

The samples were conditioned in a climatic chamber 20 °C and 65% relative humidity, the wet mass was recorded and they were dried in an oven to obtain the dry mass. The equilibrium moisture content was calculated according to the equation, EMC (%) = [(WM—DM)/DM) * 100], where EMC (%) is the equilibrium moisture content, WM = wet mass, and DM = Dry mass.

The apparent density (g/cm^3^) was determined by direct measurement of all samples placed in a climate chamber (20 °C and 60% relative humidity) dividing their mass (g) by the corresponding volume (cm^3^).

The higher heating value was obtained according to EN 14,918^56^, using a bomb calorimeter.

The structural, immediate and elemental chemical composition, according to TAPPI standards, were obtained from untreated and torrefied MDF samples, after being crushed and sieved in 40 to 60 mesh sieves.

The extractives content was determined in duplicates, according to the standard TAPPI 204 om-88^57^, changing ethanol/benzene for ethanol/toluene. The insoluble lignin content was determined in duplicate by Klason method^58^. Soluble lignin was determined by spectrometry^59^. The sum of the soluble and insoluble lignin values allowed obtaining the total lignin content. The holocellulose content (cellulose and hemicellulose) was determined by the sum of the extractive content, total lignin and ash content, decreased by 100.

The immediate chemical composition of the biomasses (volatile materials, ash content and fixed carbon) was evaluated according to NBR 8112 standard^60^.

The elemental composition (carbon, nitrogen, hydrogen and sulfur) was determined according to the standard EN 15,104^61^. The oxygen content was obtained by adding the contents of carbon, nitrogen, hydrogen, sulfur and ashes decreased by 100, according to the standard EN 15,296^62^.

### Apparent density and densitometric profiles by X-ray method

MDF samples (18 × 18 × 2 mm, length x width x thickness), in all treatments, were cut with a circular saw mill and placed in a climate chamber (20 °C, 60% relative humidity and 12% wood moisture) for twenty-four hours^22^.

The untreated and torrefied MDF samples with 2 mm thickness were inserted with the cellulose acetate calibration scale in the armored compartment of the digital X-ray equipment Faxitron LX-60 calibrated for automatic reading (30 kV, 19 s). The digital images, with ultra-contrast and resolution, were saved in the DICOM format (64). The apparent density profiles were obtained with digital gray scale images and the calibration was analyzed using the ImageJ software. This allowed determining the apparent density values (every 50 μm) obtained by the software and transferred to the spreadsheet. From the digital images in gray scale, they were transformed into a rainbow scale, with the Photoshop software, and the plotting of the 3D surface of density variation, with the ImageJ software.

### Statistical analysis

The results of the energetic characterization, in relation to the torrefaction time of the MDF samples, were analyzed in a completely randomized design with four treatments (untreated and three torrefaction times) and six replications per parameter. Five samples from each treatment were used for the analysis of apparent density (average, minimum and maximum) by digital X-ray images. The means were grouped using the Tukey test (p ≤ 0.05) with the STATISTICA 8.0 software.
